# Complete plastome sequence of *Rumex japonicus* (Polygonaceae) in Dok-do Island, Korea

**DOI:** 10.1080/23802359.2019.1660274

**Published:** 2019-09-03

**Authors:** Jin-Suk Youn, JiYoung Yang, Seung-Chul Kim, Jae-Hong Pak

**Affiliations:** a Research Institute for Dok-do and Ulleung-do Island and Department of Biology, School of Life Sciences, Kyungpook National University, Daegu, Republic of Korea;; b Department of Biological Sciences, Sungkyunkwan University, Suwon, Gyeonggi-do, Republic of Korea

**Keywords:** Chloroplast genome, Dok-do Island, *Rumex japonicus*, Polygonaceae

## Abstract

We reported the complete chloroplast genome sequence of *Rumex japonicus* in Dok-do Island, Korea. The genome size was 159,223 bp in total length with one large single-copy (LSC; 84,962 bp), one small single-copy (SSC; 12,999 bp), and 2 inverted repeat (IR) regions (IR_a_ and IR_b_, each with 30,631 bp). The overall GC content was 37.5% and the genome contained 131 genes, including 83 protein-coding, 38 transfer RNA and 8 ribosomal RNA genes. Phylogenetic analysis supported monophyly of subgenus *Rumex* and determined the phylogenetic position of *R. japonicus* as sister to the clade containing *R. crispus* and *R. nepalensis*.

The docks and sorrels genus *Rumex* L. (Polygonaceae) is known for its diverse reproductive systems and sex-determining mechanism and comprises approximately 200 species distributed worldwide (Löve and Kapoor 1967; Mosyakin 2005; Navajas-Pérez et al. 2005). It is also known as one of the richest genera in natural hybrids, especially within the subgenus *Rumex* (Rechinger 1984; Daehler and Carino 2001). Given its large number of species and frequent hybridization, the genus *Rumex* has had a complicated taxonomic history, resulted in various infrageneric classification systems (e.g., Meisner 1856; Willkomm 1862; Dammer 1893; Rechinger 1964; Löve and Kapoor 1967). The molecular phylogenetic study of *Rumex sensu lato* recognized two large clades; one formed by the species of subgenus *Rumex* and the other composed of the species of subgenera *Acetosa*, *Acetosella*, and *Platypodium* (Navajas-Pérez et al. 2005). *Rumex japoincus* Houtt. is widely distributed in the Northeast Asia (China, Japan, and Korea), including Dok-do Island, which is located about 87.4 km away from Ulleung-do Island in East Sea. The Dok-do is oceanic volcanic island, consisting of two main islets (Seo-do; 88,740 m^2^ and Dong-do; 73,300 m^2^) and 89 annexed islets (the total area of 187,554 m^2^) (Sohn 1995). The Dok-do Island was formed from the early to late Pliocene (Sohn 1995). In Dok-do, about 60 native and introduced vascular plant species occur, one of which is *R. japoincus*, the most widely distributed species in both islets (Jung et al. 2014).

We sequenced the complete plastome of *R. japonicus* sampled from Dok-do Island (Voucher specimen; 37°14′26″ 131°52′10″, KNU-YJS20170714) to develop molecular markers for species identification and conduct phylogenetic and population genetic studies. Total DNA was isolated using the DNeasy plant Mini Kit (Qiagen, Carlsbad, CA, USA) and sequenced by the Illumina platform (Macrogen, Seoul, Korea). A total of 50,101,414 paired-end reads were obtained and assembled *de nove* with Velvet v. 1.2.10 using multiple k-mers (Zerbion and Birney 2008). The tRNAs were confirmed using tRNAscan-SE (Lower and Eddy 1997). The complete plastome of *R*. *japonicus* (MK058527) was 159,223 bp in total length with one large single-copy region (LSC; 84,962 bp), one small single-copy region (SSC; 12,999 bp), and two inverted repeat regions (IR_a_ and IR_b_; 30,631 bp each). The overall GC content was 37.5% (LSC, 35.6%; SSC, 32.6%; IRs, 41.1%). The plastome contained 131 genes, including 83 protein-coding, 38 tRNA and 8 rRNA genes. The pairs of *rpl*23 gene located in IRs became pseudogene. The 16 genes contained one intron, while two genes (*clp*P and *ycf*3) contained two introns. A total of 18 genes were duplicated in IRs, including 7 tRNA, 4 rRNA, 6 protein-coding genes, and 1 pseudogene. Phylogenetic analysis of 13 representative species in the family Polygonaceae and one outgroup species (*Plumbago auriculata*, NC041245; Plumbaginaceae) was conducted using IQ-TREE v.1.4.2 (Nguyen et al. 2015) based on MAFFT v.7 (Katoh and Standley 2013) alignment. The ML tree (Figure 1) suggested that subgenus *Rumex* is monophyletic (100% bootstrap support) and that *R. japonicus* is sister to the clade containing *R. nepalensis* and *R. crispus*.

**Figure 1.**
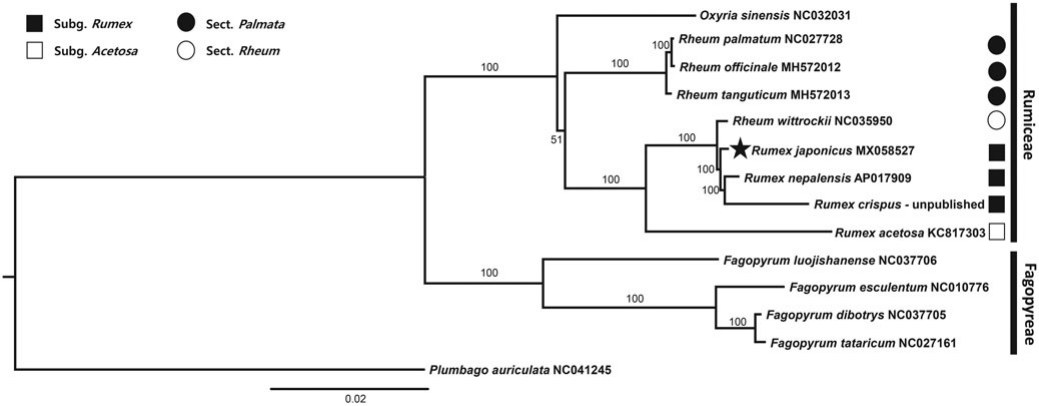
The maximum-likelihood (ML) tree based on 13 representatives of Polygonaceae and one ougroup taxon, *Plumbago auriculata* (Plumbaginaceae). The bootstrap support value based on 1000 replicates is shown on each node.
